# Benzene-1,2,4,5-tetrol

**DOI:** 10.1107/S2414314624006126

**Published:** 2024-06-28

**Authors:** Benjamin L. Weare, Sean Hoggett, William J. Cull, Stephen P. Argent, Andrei N. Khlobystov, Paul D. Brown

**Affiliations:** a Nanoscale and Microscale Research Centre, University of Nottingham, Nottingham, NG7 2RD, United Kingdom; b School of Chemistry, University of Nottingham, Nottingham, NG7 2RD, United Kingdom; c Department of Mechanical, Materials, & Manufacturing Engineering, Faculty of Engineering, University of Nottingham, Nottingham, NG7 2RD, United Kingdom; University of Antofagasta, Chile

**Keywords:** benzene-1,2,4,5-tetrol, crystal structure, hydrogen bonds, covalent organic framework, hydrox­yl

## Abstract

The crystal structure of the title compound was determined at 120 K. It crystallizes in the triclinic space group *P* with four independent mol­ecules in the asymmetric unit. In the crystal, each symmetry-unique mol­ecule forms π–π stacks on itself, giving four unique π–π stacking inter­actions. Inter­molecular hydrogen bonding is observed between each pair of independent mol­ecules, where each hy­droxy group can act as a hydrogen-bond donor and acceptor.

## Abstract 2

Determination of the structure of benzene-1,2,4,5-tetrol

## Graphical abstract

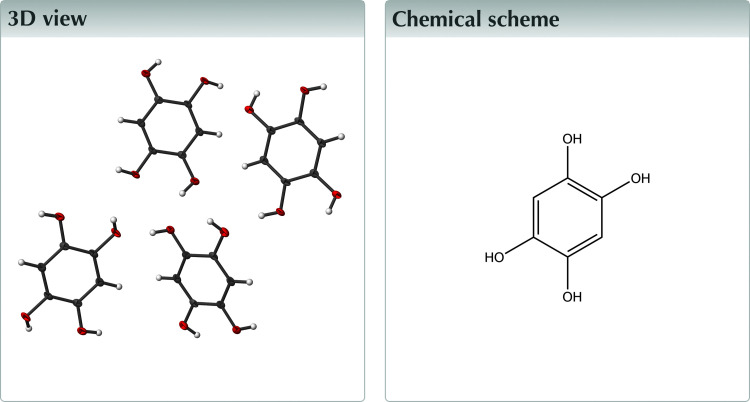

## Structure description

Benzene-1,2,4,5-tetrol, a derivative of 2,5-dihy­droxy-1,4-benzo­quinone, has seen extensive use as a precursor to functionalized benzenes as well as more complex mol­ecules and ligands. It has been used to access a number of more complex organic structures, such as phospho­rous-containing ligands for transition-metal complexes (Pandey *et al.*, 2019 ▸) or to bridge metal centres in complexes (Wellala *et al.*, 2018 ▸). In recent years benzene-1,2,4,5-tetrol has found a niche as a monomer for the synthesis of polymers, coordination polymers, covalent organic frameworks, and a variety of other supra­molecular structures. It has seen extensive use in the synthesis of framework polymers where it acts as a linear monomer linking other structural units. Recent examples include combining benzene-1,2,4,5-tetrol with a boronic acid-containing porphyrin, a two-dimensional square-pored boronate ester covalent organic framework (COF), creating a thin film that could be integrated into a field-effect transistor (Park *et al.*, 2020 ▸), as well as the creation of hafnium- and zirconium-containing coordination polymers with water sorption properties, using benzene-1,2,4,5-tetrol as a linker (Poschmann *et al.*, 2021 ▸). Benzene-1,2,4,5-tetrol has also been used in the synthesis of a variety of other COFs (Rondelli *et al.*, 2023 ▸; Dalapati *et al.*, 2015 ▸; Ma *et al.*, 2013 ▸; Lanni *et al.*, 2011 ▸), coordination polymers (Abrahams *et al.*, 2016 ▸), supra­molecular structures (Jia *et al.*, 2015 ▸; Niu *et al.*, 2006 ▸; Nakabayashi & Ohkoshi, 2009 ▸; Yuan *et al.*, 2012 ▸), and polymers (Christinat *et al.*, 2007 ▸; Rambo & Lavigne, 2007 ▸; Nishiyabu *et al.*, 2012 ▸).

Despite of the ongoing inter­est in benzene-1,2,4,5-tetrol as a reagent, which stretches back at least a century (Mukerji, 1922 ▸), the crystal structure has only been solved as a water solvate and a co-crystal with 2,5-dihy­droxy-1,4-benzo­quinone (Jene *et al.*, 2001 ▸). A search of the Cambridge Structure Database (WebCSD, December 2023) for the mol­ecular structure of 1,2,4,5-benzene­tetrol gave three results: 1,2,4,5-tetra­hydroxy­benzene monohydrate (QOGMAA; Jene *et al.*, 2001 ▸); and 1,2,4,5-tetra­hydroxy­benzene 2,5-dihy­droxy-1,4-benzo­quinone (QOGMII, QOGMII01; Jene *et al.*, 2001 ▸). Here we present the crystal structure of benzene-1,2,4,5-tetrol for the first time, which we anti­cipate will be of use for the synthetic chemical community in future endeavours.

**Figure 1:**
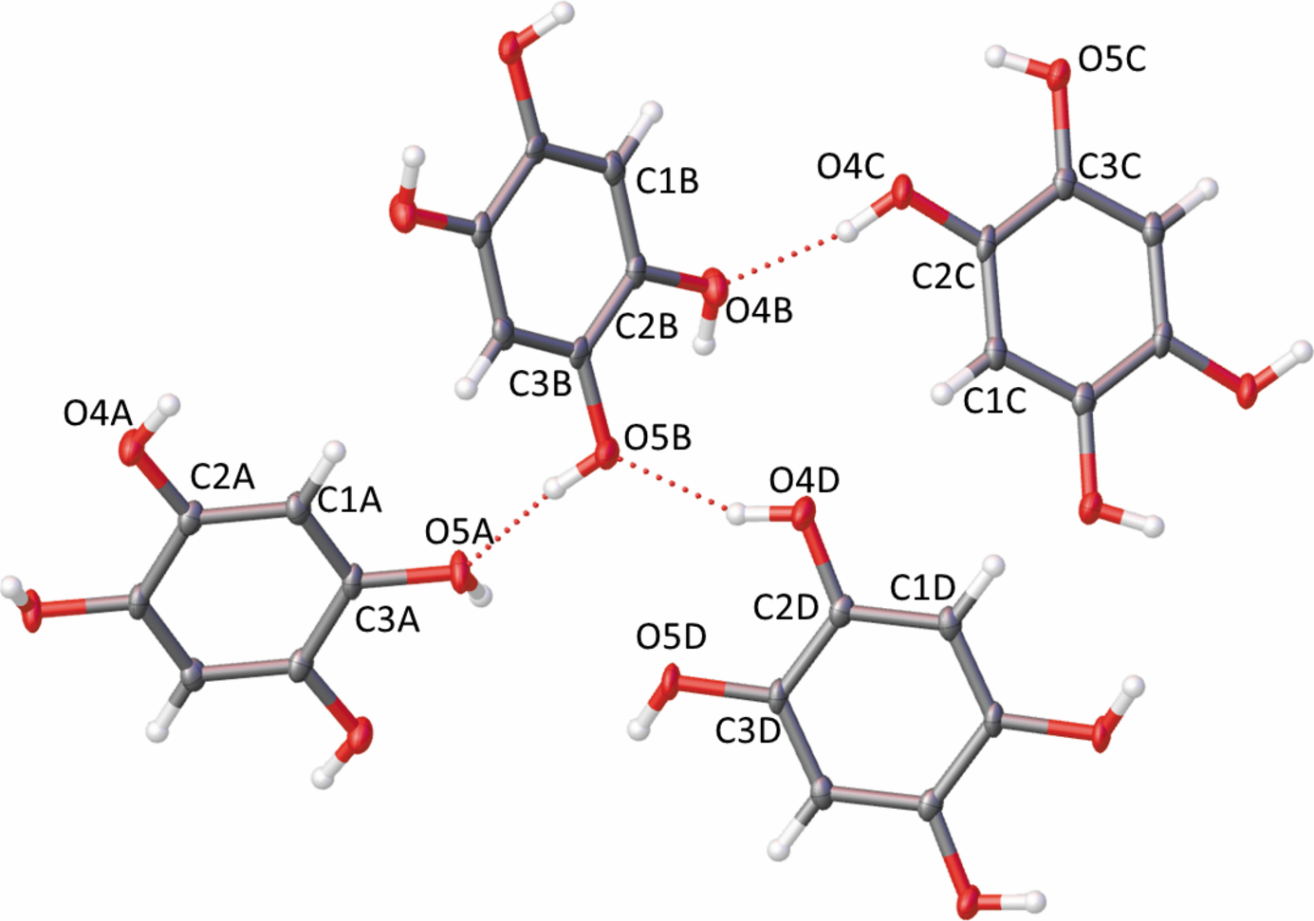
The asymmetric unit of the title compound showing the atom labelling with 50% probability displacement ellipsoids. Unlabelled atoms are related to labelled atoms by the symmetry operations −*x*, −*y* + 2, −*z* for mol­ecule *A*, −*x* + 1, −*y* + 1, −*z* for mol­ecule *B*, −*x* + 1, −*y*, −*z* + 1 for mol­ecule *C* and −*x* + 2, −*y* + 1, −*z* + 1 for mol­ecule *D*.

**Figure 2:**
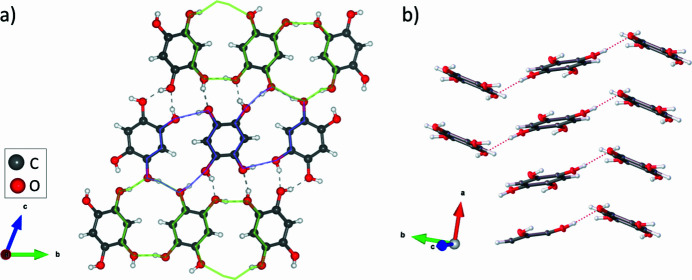
(*a*) View of unit cell along the crystallographic *a*-axis. Dashed lines represent hydrogen bonding between mol­ecules. 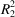
(14) rings are indicated with purple and green polygons; hydrogen bonds not lying on the indicated rings form the same class of ring with mol­ecules not rendered in this diagram. (*b*) View approximately along the (001) axis, showing how mol­ecules form π–π stacks. Some mol­ecules have been removed for clarity.

At 120 K the structure was found to crystallize in the triclinic space group *P*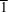 with the asymmetric unit containing four independent mol­ecules of benzene-1,2,4,5-tetrol labelled *A*, *B*, *C* and *D* (Figs. 1 ▸, 2*a* ▸). Each symmetry unique mol­ecule forms π–π stacks on itself, *i.e.* mol­ecule *A* forms a stack consisting entirely of mol­ecule *A* (Fig. 2 ▸*b*). This gives four unique π–π stacking inter­actions with centroid-to-distances of 3.7474 (11) Å, while the perpendicular centroid-to-plane distances are 3.4457 (7) Å (mol­ecule *A*), 3.5166 (8) Å (mol­ecule *B*), 3.5653 (8) Å (mol­ecule *C*), and 3.5653 (8) Å (mol­ecule *D*). Inter­molecular hydrogen bonding is observed between each pair of mol­ecules, where each hy­droxy group can act as a hydrogen-bond donor and acceptor (Table 1 ▸). This creates an extended hydrogen-bond network, which can be described as a series of rings consisting of three mol­ecules – the edges of two mol­ecules make up the perimeter of the ring, and a single hy­droxy group of a third mol­ecule links the first two mol­ecules into a continuous ring. There are two unique rings comprised of mol­ecules *A*, *B*, and *C*, and of mol­ecules *C*, *B*, and *D*, both of which exhibit an 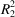
(14) graph-set motif, and the remaining hydrogen-bonded rings are symmetry-related. All of the hydrogen bonds in the structure can thus be accounted for.

## Synthesis and crystallization

Following a literature procedure (Weider *et al.*, 1985 ▸), 2,5-dihy­droxy-1,4-benzo­quinone (2.428 g, 17.3 mmol) was mixed with conc. hydro­chloric acid (54 ml) under an inert atmosphere and stirred for 30 min to form a gold-coloured suspension. Addition of tin metal powder (2.1885 g, 18.4 mmol) caused vigorous effervescence and a grey suspension. The mixture was stirred for 10 min until cessation of bubbling then heated to 100° C for 1 h, during which time the mixture became dark and bubbled vigorously. The mixture was allowed to cool briefly, then hot filtered under reduced pressure to give a yellow filtrate. The filtrate was cooled on ice for 30 min to give white crystals of benzene-1,2,4,5-tetrol (0.786 g, 5.54 mmol, 32%). The crude product was dissolved in a minimum of hot tetra­hydro­furan, filtered, then cooled on ice. The resulting white crystals were collected *via* filtration then washed with ice-cold THF and dried in a vacuum to give benzene-1,2,4,5-tetrol (0.735 g, 5.17 mmol, 30%). IR (ATR) ν_max_ /cm^−1^: 3146.01 *br* (OH), 1551.54 *s* (Ar C—C), 1155.90 *w* (C—O) MS (ESI) *m*/*z*: 165.02 (*M*+Na). ^1^H NMR (400 MHz, DMSO-*d*_6_, p.p.m., δ): 9.66 (*s*, 4H, OH), 5.94 (*s*, 2H, Ar H); ^13^C NMR (400 MHz, DMSO-*d*_6_, p.p.m., δ): 138.46, 104.81. CNH analysis found: C, 50.6; H, 4.1; N, 0. Calculated for C_6_H_6_O_4_: C, 50.7; H, 4.3; N, 0%.

## Refinement

Crystal data, data collection and structure refinement details are summarized in Table 2 ▸.

## Supplementary Material

Crystal structure: contains datablock(s) I. DOI: 10.1107/S2414314624006126/bx4030sup1.cif

Structure factors: contains datablock(s) I. DOI: 10.1107/S2414314624006126/bx4030Isup2.hkl

Supporting information file. DOI: 10.1107/S2414314624006126/bx4030Isup3.cml

CCDC reference: 2357698

Additional supporting information:  crystallographic information; 3D view; checkCIF report

## Figures and Tables

**Table 1:** Hydrogen-bond geometry (Å, °)

*D*—H⋯*A*	*D*—H	H⋯*A*	*D*⋯*A*	*D*—H⋯*A*
O4*A*—H4*A*⋯O4*C*^i^	0.85 (2)	1.89 (2)	2.715 (2)	163 (2)
O4*B*—H4*B*⋯O4*D*^ii^	0.86 (2)	1.88 (2)	2.708 (2)	163 (3)
O4*B*—H4*B*⋯O5*B*	0.86 (2)	2.45 (2)	2.764 (2)	102 (2)
O4*C*—H4*C*⋯O4*B*	0.86 (2)	1.85 (2)	2.702 (2)	167 (2)
O4*D*—H4*D*⋯O5*B*	0.86 (2)	1.85 (2)	2.6425 (19)	154 (2)
O4*D*—H4*D*⋯O5*D*	0.86 (2)	2.34 (2)	2.789 (2)	113 (2)
O5*A*—H5*A*⋯O4*A*	0.83 (2)	2.40 (2)	2.711 (2)	103 (2)
O5*A*—H5*A*⋯O5*D*^ii^	0.83 (2)	1.95 (2)	2.7562 (18)	162 (2)
O5*B*—H5*B*⋯O5*A*	0.84 (2)	1.80 (2)	2.633 (2)	169 (2)
O5*C*—H5*C*⋯O4*A*^iii^	0.83 (2)	2.04 (2)	2.8376 (16)	161 (2)
O5*C*—H5*C*⋯O4*C*	0.83 (2)	2.38 (2)	2.734 (2)	107 (2)
O5*D*—H5*D*⋯O5*C*^iv^	0.85 (2)	2.03 (2)	2.8796 (19)	175 (2)

Symmetry codes: (i) 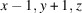; (ii) 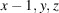; (iii) 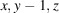; (iv) 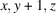.

**Table 2:** Experimental details

Crystal data	
Chemical formula	C_6_H_6_O_4_
*M*_r_	142.11
Crystal system, space group	Triclinic, *P* 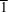
Temperature (K)	120
*a*, *b*, *c* (Å)	3.7474 (2), 11.6254 (6), 13.7771 (8)
α, β, γ (°)	68.407 (5), 85.779 (4), 89.843 (4)
*V* (Å^3^)	556.37 (6)
*Z*	4
Radiation type	Cu *K*α
μ (mm^−1^)	1.27
Crystal size (mm)	0.07 × 0.05 × 0.02

Data collection	
Diffractometer	XtalLAB PRO MM007, PILATUS3 R 200K
Absorption correction	Gaussian (*CrysAlis PRO*; Rigaku OD, 2023 ▸)
*T*_min_, *T*_max_	0.927, 1.000
No. of measured, independent and observed [*I* > 2σ(*I*)] reflections	8096, 2185, 1842
*R*_int_	0.063
(sin θ/λ)_max_ (Å^−1^)	0.630

Refinement	
*R*[*F*^2^ > 2σ(*F*^2^)], *wR*(*F*^2^), *S*	0.045, 0.133, 1.09
No. of reflections	2185
No. of parameters	205
No. of restraints	8
H-atom treatment	H atoms treated by a mixture of independent and constrained refinement
Δρ_max_, Δρ_min_ (e Å^−3^)	0.28, −0.34

Computer programs: *CrysAlis PRO* (Rigaku OD, 2023 ▸), *SHELXT2018/2* (Sheldrick, 2015*a* ▸), *SHELXL2018/3* (Sheldrick, 2015*b* ▸) and *OLEX2* (Dolomanov *et al.*, 2009 ▸).

## full crystallographic data

### Benzene-1,2,4,5-tetrol Crystal data

**Table:**

C_6_H_6_O_4_	*Z* = 4
*M**_r_* = 142.11	*F*(000) = 296
Triclinic, *P*1	*D*_x_ = 1.697 Mg m^−^^3^
*a* = 3.7474 (2) Å	Cu *K*α radiation, λ = 1.54184 Å
*b* = 11.6254 (6) Å	Cell parameters from 4683 reflections
*c* = 13.7771 (8) Å	θ = 3.4–75.6°
α = 68.407 (5)°	µ = 1.26 mm^−^^1^
β = 85.779 (4)°	*T* = 120 K
γ = 89.843 (4)°	Block, colourless
*V* = 556.37 (6) Å^3^	0.07 × 0.05 × 0.02 mm

### Benzene-1,2,4,5-tetrol Data collection

**Table:**

XtalLAB PRO MM007, PILATUS3 R 200K diffractometer	2185 independent reflections
Radiation source: rotating anode, MicroMax 007 HF	1842 reflections with *I* > 2σ(*I*)
Mirror monochromator	*R*_int_ = 0.063
Detector resolution: 5.8140 pixels mm^-1^	θ_max_ = 76.2°, θ_min_ = 3.5°
ω scans	*h* = −4→4
Absorption correction: gaussian (CrysAlisPro; Rigaku OD, 2023)	*k* = −14→14
*T*_min_ = 0.927, *T*_max_ = 1.000	*l* = −17→17
8096 measured reflections	

### Benzene-1,2,4,5-tetrol Refinement

**Table:**

Refinement on *F*^2^	Primary atom site location: dual
Least-squares matrix: full	Secondary atom site location: difference Fourier map
*R*[*F*^2^ > 2σ(*F*^2^)] = 0.045	Hydrogen site location: mixed
*wR*(*F*^2^) = 0.133	H atoms treated by a mixture of independent and constrained refinement
*S* = 1.09	*w* = 1/[σ^2^(*F*_o_^2^) + (0.0799*P*)^2^ + 0.1541*P*] where *P* = (*F*_o_^2^ + 2*F*_c_^2^)/3
2185 reflections	(Δ/σ)_max_ < 0.001
205 parameters	Δρ_max_ = 0.28 e Å^−^^3^
8 restraints	Δρ_min_ = −0.33 e Å^−^^3^

### Benzene-1,2,4,5-tetrol Special details

**Table:**

Geometry. All esds (except the esd in the dihedral angle between two l.s. planes) are estimated using the full covariance matrix. The cell esds are taken into account individually in the estimation of esds in distances, angles and torsion angles; correlations between esds in cell parameters are only used when they are defined by crystal symmetry. An approximate (isotropic) treatment of cell esds is used for estimating esds involving l.s. planes.
Refinement. All hydrogen atoms were observed in the electron difference map. All hydroxy hydrogen atoms were refined with their O-H distances restrained to a target distance of 0.84 %A (DFIX). All other hydrogen atoms were geometrically placed and refined with a riding model.

### Benzene-1,2,4,5-tetrol Fractional atomic coordinates and isotropic or equivalent isotropic displacement parameters (Å^2^)

**Table:**

	*x*	*y*	*z*	*U*_iso_*/*U*_eq_	
C1A	−0.1468 (5)	1.09568 (17)	0.02598 (13)	0.0161 (4)	
H1A	−0.249116	1.160936	0.043654	0.019*	
C2A	−0.0561 (4)	0.98695 (17)	0.10428 (13)	0.0161 (4)	
C3A	0.0876 (5)	0.89114 (17)	0.07847 (13)	0.0159 (4)	
O4A	−0.1011 (3)	0.96812 (13)	0.20910 (9)	0.0204 (3)	
H4A	−0.224 (6)	1.024 (2)	0.2205 (19)	0.031*	
O5A	0.1865 (4)	0.78369 (12)	0.15524 (10)	0.0223 (3)	
H5A	0.055 (6)	0.767 (2)	0.2104 (15)	0.033*	
C1B	0.3702 (5)	0.38074 (17)	0.02176 (14)	0.0176 (4)	
H1B	0.280395	0.299234	0.036481	0.021*	
C2B	0.3851 (5)	0.42502 (17)	0.10209 (13)	0.0164 (4)	
C3B	0.5136 (5)	0.54465 (17)	0.08035 (14)	0.0167 (4)	
O4B	0.2720 (4)	0.34746 (13)	0.20260 (10)	0.0231 (3)	
H4B	0.192 (7)	0.388 (2)	0.2403 (18)	0.035*	
O5B	0.5337 (4)	0.58394 (13)	0.16233 (10)	0.0225 (3)	
H5B	0.434 (7)	0.6521 (18)	0.1517 (19)	0.034*	
C1C	0.5825 (5)	0.12616 (17)	0.45145 (13)	0.0168 (4)	
H1C	0.639580	0.212043	0.418210	0.020*	
C2C	0.4847 (5)	0.05791 (17)	0.39276 (13)	0.0167 (4)	
C3C	0.4038 (5)	−0.06764 (17)	0.44063 (13)	0.0161 (4)	
O4C	0.4713 (4)	0.10954 (12)	0.28510 (9)	0.0195 (3)	
H4C	0.421 (6)	0.1868 (16)	0.2668 (18)	0.029*	
O5C	0.3112 (3)	−0.13754 (12)	0.38383 (9)	0.0185 (3)	
H5C	0.226 (6)	−0.093 (2)	0.3286 (14)	0.028*	
C1D	1.0908 (4)	0.38787 (17)	0.49271 (13)	0.0159 (4)	
H1D	1.153969	0.310947	0.487689	0.019*	
C2D	0.9455 (4)	0.47869 (17)	0.40886 (13)	0.0149 (4)	
C3D	0.8562 (4)	0.59139 (17)	0.41683 (13)	0.0151 (4)	
O4D	0.8906 (4)	0.45066 (13)	0.32286 (9)	0.0202 (3)	
H4D	0.773 (6)	0.509 (2)	0.2812 (17)	0.030*	
O5D	0.7198 (3)	0.68082 (12)	0.33076 (9)	0.0180 (3)	
H5D	0.611 (6)	0.7358 (19)	0.3477 (18)	0.027*	

### Benzene-1,2,4,5-tetrol Atomic displacement parameters (Å^2^)

**Table:**

	*U*^11^	*U*^22^	*U*^33^	*U*^12^	*U*^13^	*U*^23^
C1A	0.0121 (8)	0.0164 (9)	0.0193 (9)	0.0051 (7)	−0.0046 (7)	−0.0052 (7)
C2A	0.0106 (8)	0.0201 (10)	0.0156 (8)	0.0037 (7)	−0.0036 (6)	−0.0039 (7)
C3A	0.0126 (8)	0.0159 (9)	0.0153 (8)	0.0037 (7)	−0.0049 (6)	−0.0003 (7)
O4A	0.0238 (7)	0.0217 (7)	0.0146 (6)	0.0096 (6)	−0.0051 (5)	−0.0046 (5)
O5A	0.0267 (7)	0.0185 (7)	0.0153 (6)	0.0120 (6)	−0.0023 (5)	0.0012 (5)
C1B	0.0142 (8)	0.0154 (9)	0.0215 (9)	0.0067 (7)	−0.0072 (7)	−0.0036 (7)
C2B	0.0128 (8)	0.0166 (9)	0.0162 (8)	0.0056 (7)	−0.0055 (7)	−0.0009 (7)
C3B	0.0136 (8)	0.0191 (9)	0.0183 (8)	0.0092 (7)	−0.0096 (7)	−0.0065 (7)
O4B	0.0302 (8)	0.0182 (7)	0.0171 (6)	0.0073 (6)	−0.0010 (5)	−0.0022 (5)
O5B	0.0309 (8)	0.0196 (7)	0.0195 (7)	0.0136 (6)	−0.0128 (6)	−0.0083 (6)
C1C	0.0143 (8)	0.0162 (9)	0.0172 (8)	0.0063 (7)	−0.0036 (7)	−0.0024 (7)
C2C	0.0130 (8)	0.0194 (10)	0.0133 (8)	0.0072 (7)	−0.0039 (6)	−0.0005 (7)
C3C	0.0119 (8)	0.0176 (9)	0.0171 (8)	0.0058 (7)	−0.0040 (6)	−0.0041 (7)
O4C	0.0264 (7)	0.0157 (7)	0.0138 (6)	0.0074 (6)	−0.0068 (5)	−0.0013 (5)
O5C	0.0210 (7)	0.0175 (7)	0.0154 (6)	0.0051 (6)	−0.0080 (5)	−0.0032 (5)
C1D	0.0123 (8)	0.0145 (9)	0.0183 (8)	0.0043 (7)	−0.0036 (7)	−0.0026 (7)
C2D	0.0103 (8)	0.0180 (9)	0.0148 (8)	0.0026 (7)	−0.0033 (6)	−0.0037 (7)
C3D	0.0120 (8)	0.0144 (9)	0.0150 (8)	0.0041 (7)	−0.0045 (6)	0.0000 (7)
O4D	0.0237 (7)	0.0210 (7)	0.0164 (6)	0.0107 (6)	−0.0094 (5)	−0.0062 (5)
O5D	0.0194 (6)	0.0171 (7)	0.0148 (6)	0.0092 (5)	−0.0075 (5)	−0.0015 (5)

### Benzene-1,2,4,5-tetrol Geometric parameters (Å, º)

**Table:**

C1A—H1A	0.9500	C1C—H1C	0.9500
C1A—C2A	1.388 (2)	C1C—C2C	1.391 (3)
C1A—C3A^i^	1.391 (2)	C1C—C3C^iii^	1.394 (2)
C2A—C3A	1.385 (3)	C2C—C3C	1.386 (3)
C2A—O4A	1.376 (2)	C2C—O4C	1.385 (2)
C3A—O5A	1.378 (2)	C3C—O5C	1.379 (2)
O4A—H4A	0.852 (17)	O4C—H4C	0.863 (17)
O5A—H5A	0.835 (17)	O5C—H5C	0.832 (16)
C1B—H1B	0.9500	C1D—H1D	0.9500
C1B—C2B	1.386 (2)	C1D—C2D	1.392 (2)
C1B—C3B^ii^	1.391 (3)	C1D—C3D^iv^	1.382 (2)
C2B—C3B	1.390 (3)	C2D—C3D	1.392 (3)
C2B—O4B	1.381 (2)	C2D—O4D	1.368 (2)
C3B—O5B	1.372 (2)	C3D—O5D	1.3881 (19)
O4B—H4B	0.861 (16)	O4D—H4D	0.858 (16)
O5B—H5B	0.843 (17)	O5D—H5D	0.851 (16)
			
C2A—C1A—H1A	120.1	C2C—C1C—H1C	120.2
C2A—C1A—C3A^i^	119.82 (17)	C2C—C1C—C3C^iii^	119.53 (18)
C3A^i^—C1A—H1A	120.1	C3C^iii^—C1C—H1C	120.2
C3A—C2A—C1A	120.03 (16)	C3C—C2C—C1C	120.55 (16)
O4A—C2A—C1A	123.07 (16)	O4C—C2C—C1C	122.61 (17)
O4A—C2A—C3A	116.90 (15)	O4C—C2C—C3C	116.82 (16)
C2A—C3A—C1A^i^	120.14 (16)	C2C—C3C—C1C^iii^	119.92 (17)
O5A—C3A—C1A^i^	119.11 (16)	O5C—C3C—C1C^iii^	118.51 (17)
O5A—C3A—C2A	120.70 (15)	O5C—C3C—C2C	121.57 (15)
C2A—O4A—H4A	112.3 (16)	C2C—O4C—H4C	109.5 (15)
C3A—O5A—H5A	111.3 (17)	C3C—O5C—H5C	110.6 (17)
C2B—C1B—H1B	119.9	C2D—C1D—H1D	119.7
C2B—C1B—C3B^ii^	120.17 (18)	C3D^iv^—C1D—H1D	119.7
C3B^ii^—C1B—H1B	119.9	C3D^iv^—C1D—C2D	120.58 (17)
C1B—C2B—C3B	119.88 (17)	C1D—C2D—C3D	119.20 (16)
O4B—C2B—C1B	118.40 (17)	O4D—C2D—C1D	117.50 (16)
O4B—C2B—C3B	121.71 (16)	O4D—C2D—C3D	123.28 (15)
C2B—C3B—C1B^ii^	119.95 (17)	C1D^iv^—C3D—C2D	120.22 (15)
O5B—C3B—C1B^ii^	121.84 (18)	C1D^iv^—C3D—O5D	122.17 (16)
O5B—C3B—C2B	118.17 (16)	O5D—C3D—C2D	117.60 (15)
C2B—O4B—H4B	111.8 (18)	C2D—O4D—H4D	108.3 (17)
C3B—O5B—H5B	112.5 (16)	C3D—O5D—H5D	111.5 (16)
			
C1A—C2A—C3A—C1A^i^	1.1 (3)	C1C—C2C—C3C—C1C^iii^	0.5 (3)
C1A—C2A—C3A—O5A	178.53 (16)	C1C—C2C—C3C—O5C	−179.04 (15)
C3A^i^—C1A—C2A—C3A	−1.0 (3)	C3C^iii^—C1C—C2C—C3C	−0.5 (3)
C3A^i^—C1A—C2A—O4A	178.86 (16)	C3C^iii^—C1C—C2C—O4C	−178.89 (15)
O4A—C2A—C3A—C1A^i^	−178.86 (16)	O4C—C2C—C3C—C1C^iii^	178.98 (15)
O4A—C2A—C3A—O5A	−1.4 (3)	O4C—C2C—C3C—O5C	−0.6 (2)
C1B—C2B—C3B—C1B^ii^	−0.5 (3)	C1D—C2D—C3D—C1D^iv^	−0.4 (3)
C1B—C2B—C3B—O5B	−178.19 (14)	C1D—C2D—C3D—O5D	178.51 (15)
C3B^ii^—C1B—C2B—C3B	0.5 (3)	C3D^iv^—C1D—C2D—C3D	0.4 (3)
C3B^ii^—C1B—C2B—O4B	−179.07 (15)	C3D^iv^—C1D—C2D—O4D	−178.16 (16)
O4B—C2B—C3B—C1B^ii^	179.06 (15)	O4D—C2D—C3D—C1D^iv^	178.07 (17)
O4B—C2B—C3B—O5B	1.4 (2)	O4D—C2D—C3D—O5D	−3.0 (3)

Symmetry codes: (i) −*x*, −*y*+2, −*z*; (ii) −*x*+1, −*y*+1, −*z*; (iii) −*x*+1, −*y*, −*z*+1; (iv) −*x*+2, −*y*+1, −*z*+1.

### Benzene-1,2,4,5-tetrol Hydrogen-bond geometry (Å, º)

**Table:**

*D*—H···*A*	*D*—H	H···*A*	*D*···*A*	*D*—H···*A*
O4*A*—H4*A*···O4*C*^v^	0.85 (2)	1.89 (2)	2.715 (2)	163 (2)
O4*B*—H4*B*···O4*D*^vi^	0.86 (2)	1.88 (2)	2.708 (2)	163 (3)
O4*B*—H4*B*···O5*B*	0.86 (2)	2.45 (2)	2.764 (2)	102 (2)
O4*C*—H4*C*···O4*B*	0.86 (2)	1.85 (2)	2.702 (2)	167 (2)
O4*D*—H4*D*···O5*B*	0.86 (2)	1.85 (2)	2.6425 (19)	154 (2)
O4*D*—H4*D*···O5*D*	0.86 (2)	2.34 (2)	2.789 (2)	113 (2)
O5*A*—H5*A*···O4*A*	0.83 (2)	2.40 (2)	2.711 (2)	103 (2)
O5*A*—H5*A*···O5*D*^vi^	0.83 (2)	1.95 (2)	2.7562 (18)	162 (2)
O5*B*—H5*B*···O5*A*	0.84 (2)	1.80 (2)	2.633 (2)	169 (2)
O5*C*—H5*C*···O4*A*^vii^	0.83 (2)	2.04 (2)	2.8376 (16)	161 (2)
O5*C*—H5*C*···O4*C*	0.83 (2)	2.38 (2)	2.734 (2)	107 (2)
O5*D*—H5*D*···O5*C*^viii^	0.85 (2)	2.03 (2)	2.8796 (19)	175 (2)

Symmetry codes: (v) *x*−1, *y*+1, *z*; (vi) *x*−1, *y*, *z*; (vii) *x*, *y*−1, *z*; (viii) *x*, *y*+1, *z*.

## References

[bb1] Abrahams, B. F., Dharma, A. D., Dyett, B., Hudson, T. A., Maynard-Casely, H., Kingsbury, C. J., McCormick, L. J., Robson, R., Sutton, A. L. & White, K. F. (2016). *Dalton Trans.* **45**, 1339–1344. 10.1039/c5dt04095g 26733002

[bb2] Christinat, N., Croisier, E., Scopelliti, R., Cascella, M., Röthlisberger, U. & Severin, K. (2007). *Eur. J. Inorg. Chem.* pp. 5177–5181.

[bb3] Dalapati, S., Addicoat, M., Jin, S., Sakurai, T., Gao, J., Xu, H., Irle, S., Seki, S. & Jiang, D. (2015). *Nat. Commun.* **6**, 7786. 10.1038/ncomms8786 PMC4518282 26178865

[bb4] Dolomanov, O. V., Bourhis, L. J., Gildea, R. J., Howard, J. A. K. & Puschmann, H. (2009). *J. Appl. Cryst.* **42**, 339–341.

[bb5] Jene, P. G., Pernin, C. G. & Ibers, J. A. (2001). *Acta Cryst.* C**57**, 730–734. 10.1107/s0108270101003353 11408688

[bb6] Jia, S.-H., Ding, X., Yu, H.-T. & Han, B.-H. (2015). *RSC Adv.* **5**, 71095–71101.

[bb7] Lanni, L. M., Tilford, R. W., Bharathy, M. & Lavigne, J. J. (2011). *J. Am. Chem. Soc.* **133**, 13975–13983. 10.1021/ja203807h 21806023

[bb8] Ma, H., Ren, H., Meng, S., Yan, Z., Zhao, H., Sun, F. & Zhu, G. (2013). *Chem. Commun.* **49**, 9773. 10.1039/c3cc45217d 24022638

[bb9] Mukerji, D. N. (1922). *J. Chem. Soc. Trans.* **121**, 545–552.

[bb10] Nakabayashi, K. & Ohkoshi, S. (2009). *Inorg. Chem.* **48**, 8647–8649. 10.1021/ic900625a 19685914

[bb11] Nishiyabu, R., Teraoka, S., Matsushima, Y. & Kubo, Y. (2012). *ChemPlusChem* **77**, 201–209.

[bb12] Niu, W., Smith, M. D. & Lavigne, J. J. (2006). *Cryst. Growth Des.* **6**, 1274–1277.

[bb13] Pandey, M. K., Kunchur, H. S., Ananthnag, G. S., Mague, J. T. & Balakrishna, M. S. (2019). *Dalton Trans.* **48**, 3610–3624. 10.1039/c8dt04819c 30720813

[bb14] Park, S. W., Liao, Z., Ibarlucea, B., Qi, H., Lin, H. H., Becker, D., Melidonie, J., Zhang, T., Sahabudeen, H., Baraban, L., Baek, C. K., Zheng, Z., Zschech, E., Fery, A., Heine, T., Kaiser, U., Cuniberti, G., Dong, R. & Feng, X. (2020). *Angew. Chem. Int. Ed.* **59**, 8218–8224. 10.1002/anie.201916595 PMC7317805 32039541

[bb15] Poschmann, M. P. M., Reinsch, H. & Stock, N. (2021). *Z. Anorg. Allg. Chem.* **647**, 436–441.

[bb16] Rambo, B. M. & Lavigne, J. J. (2007). *Chem. Mater.* **19**, 3732–3739.

[bb17] Rigaku OD (2023). *CrysAlis PRO*. Rigaku Oxford Diffraction, Yarnton, England.

[bb18] Rondelli, M., Daranas, A. H. & Martín, T. (2023). *J. Org. Chem.* **88**, 2113–2121. 10.1021/acs.joc.2c02523 PMC9942191 36730713

[bb19] Sheldrick, G. M. (2015*a*). *Acta Cryst.* A**71**, 3–8.

[bb20] Sheldrick, G. M. (2015*b*). *Acta Cryst.* C**71**, 3–8.

[bb21] Weider, P. R., Hegedus, L. S. & Asada, H. (1985). *J. Org. Chem.* **50**, 4276–4281.

[bb22] Wellala, N. P. N., Dong, H. T., Krause, J. A. & Guan, H. (2018). *Organometallics*, **37**, 4031–4039.

[bb23] Yuan, Y., Liu, J., Ren, H., Jing, X., Wang, W., Ma, H., Sun, F. & Zhao, H. (2012). *J. Mater. Res.* **27**, 1417–1420.

